# Cephalometric studies of the mandible, its masticatory muscles and vasculature of growing Göttingen Minipigs—A comparative anatomical study to refine experimental mandibular surgery

**DOI:** 10.1371/journal.pone.0215875

**Published:** 2019-04-25

**Authors:** Giuliano Mario Corte, Hana Hünigen, Kenneth C. Richardson, Stefan M. Niehues, Johanna Plendl

**Affiliations:** 1 Institute of Veterinary Anatomy, Department of Veterinary Medicine, Freie Universität Berlin, Berlin, Germany; 2 College of Veterinary Medicine, School of Veterinary and Life Sciences, Murdoch University, Murdoch, Western Australia; 3 Department of Radiology, Charité – Universitätsmedizin Berlin, Corporate Member of Freie Universität Berlin, Humboldt-Universität zu Berlin, and Berlin Institute of Health, Berlin, Germany; University Ceuma, BRAZIL

## Abstract

Over many decades, the Göttingen Minipig has been used as a large animal model in experimental surgical research of the mandible. Recently several authors have raised concerns over the use of the Göttingen Minipig in this research area, observing problems with post-operative wound healing and loosening implants. To reduce these complications during and after surgery and to improve animal welfare in mandibular surgery research, the present study elucidated how comparable the mandible of minipigs is to that of humans and whether these complications could be caused by specific anatomical characteristics of the minipigs’ mandible, its masticatory muscles and associated vasculature. Twenty-two mandibular cephalometric parameters were measured on CT scans of Göttingen Minipigs aged between 12 and 21 months. Ultimately, we compared this data with human data reported in the scientific literature. In addition, image segmentation was used to determine the masticatory muscle morphology and the configuration of the mandibular blood vessels. Compared to data of humans, significant differences in the mandibular anatomy of minipigs were found. Of the 22 parameters measured only four were found to be highly comparable, whilst the others were not. The 3D examinations of the minipigs vasculature showed a very prominent deep facial vein directly medial to the mandibular ramus and potentially interfering with the sectional plane of mandibular distraction osteogenesis. Damage to this vessel could result in inaccessible bleeding. The findings of this study suggest that Göttingen Minipigs are not ideal animal models for experimental mandibular surgery research. Nevertheless if these minipigs are used the authors recommend that radiographic techniques, such as computed tomography, be used in the specific planning procedures for the mandibular surgical experiments. In addition, it is advisable to choose suitable age groups and customize implants based on the mandibular dimensions reported in this study.

## Introduction

In experimental surgery, the use of the most common experimental animals worldwide i.e. mice, rats and hamsters, is limited due to their small body size. Consequently, large animal models that have closer comparability to human dimensions are needed [1]. Over recent decades, the use of primates and dogs in research, has met with increasing societal resistance, mostly on ethical grounds. However, the pig has emerged as an acceptable alternative species because it is regarded by society as a production animal [2]. Furthermore, many aspects of a pig’s physiology are similar to that of humans, making them especially suitable as large animal models for biomedical research [3–5]. Domestic pig breeds have a high adult body weight and large size that is frequently coupled with aggressive behaviour that have proven to be challenging in their husbandry [6, 7]. In 1949, the first miniature pigs namely, Minnesota minipigs, were bred to overcome these problems [8]. Subsequently since its development in the 1960s, the Göttingen Minipig has become the most widely used pig breed and one of the smallest available for research [9]. Its small size, low average adult body weight of around 35 kg and rapid growth allows easier handling and more economic housing than conventional domestic pig breeds. Furthermore, its early sexual maturity makes it more convenient for long-term studies than normal-sized pigs or other large animal models [10–13]. Because of that, the Göttingen Minipig has been used frequently in mandibular surgical research over recent decades [14, 15].

The mandible consists of two hemimandibles joined anteriorly by a symphysis that in the pig is usually ossified by 12 months of age [16]. Each hemimandible consists of a horizontal tooth-bearing mandibular body and a perpendicular mandibular ramus. The mandibular body has an anterior incisive part that contains three incisor teeth and a single canine tooth. Further posteriorly the molar part of the mandibular body houses three to four premolar and three molar teeth. A short diastema separates the incisive and molar parts of the mandible. Within the substance of the mandibular body runs the mandibular canal. This originates posteriorly at the mandibular foramen and runs anteriorly within the mandibular body to terminate immediately rostral to the mandibular molar part. The canal conveys the inferior alveolar neurovascular bundle that consists of the inferior alveolar artery, vein and nerve [17–19].

Posteriorly the mandibular ramus rises superiorly from the mandibular body. Its lateral aspect is slightly recessed forming the masseteric fossa housing a large masseteric muscle. When both left and right masseter muscles contract together, they elevate the mandible and when they contract separately they move the mandible laterally [20, 21]. The medial aspect of the ramus has a shallow recess where the medial and lateral pterygoid muscles both insert. The larger medial pterygoid muscle acts synergistically with the masseter muscle to elevate the mandible, whilst the lateral pterygoid muscle is occupied mainly with lateral movements of the mandible [21].

The posteroinferior transition of the mandibular body into the mandibular ramus forms the gonial angle. From here, the posterior border of the mandibular ramus runs nearly vertically to its free superior aspect. Here a posteriorly located condylar process connects anteriorly via a sigmoid notch, also called mandibular notch, to a much smaller coronoid process. The coronoid process is the insertion point for the temporal muscle that is partly responsible for raising the mandible. The condylar process articulates with the temporal bone, forming the temporomandibular joint [20, 21].

In many mandibular research studies, the principle of distraction osteogenesis (DO) is used in skeletal reconstruction to exploit the body’s innate capacity for bone formation in response to tensile forces. Here a distractor is fixed to the aligned bone segments to keep them in the desired plane and to separate them gradually over time at a controlled rate [22, 23]. This is performed in three stages; a latency period of several days after osteotomy which allows haematoma formation and local bridging of the gap by soft callus formation, then a slow gradual distraction to stimulate ossification during elongation, followed by a period of stable fixation allowing hard callus maturation and bone remodeling [24]. Distraction osteogenesis is a lengthy and risky procedure that can result in post-operative non-union, infection, bleeding and device failure. Any of these complications ultimately prolong the period of treatment [25].

Mandibular distraction osteogenesis (MDO) and alveolar distraction osteogenesis are among many surgical techniques that have been studied using Göttingen Minipigs [23, 26–32]. Even more important has been the search for methodologies to enhance the process of distraction by accelerating the rates of activation and bone healing or to promote the osseointegration of bony implants utilizing novel biomaterials, implant coatings, growth factors such as morphogenetic proteins, angiogenic factors and autologous mesenchymal stem cells [22, 23, 25, 33, 34].

In experimental MDO in minipigs, the osteotomy is usually performed from the superior junction of the mandibular body and ramus and extends to the inferior border of the mandible in close proximity to the mandibular angle [23]. Alveolar distraction osteogenesis is used often for the reconstruction of the alveolar bone and surrounding soft tissues to enable dental implant placement [35].

Recently several authors have raised concerns over the use of the Göttingen Minipig in dental and orofacial surgery research, observing problems with post-operative wound healing as well as loosening of implanted plates and screws [36–38]. Some authors report that the success rate of implant studies is below 60 percent [39, 40].

These situations are problematic and it is important to refine procedures to reduce these complications during and after surgery to improve animal welfare in orofacial surgery research by minimizing pain, distress and discomfort for the animals. This is in accordance to the principles of the 3Rs by Russell and Burch [41]. To fulfill these goals, it is necessary to answer the following questions [42–44]. The first being, how comparable is the mandible of minipigs to that of humans in general, and the second being, could these post-operative complications be caused by specific anatomical characteristics of the minipigs’ mandible, its masticatory muscles and associated vasculature? To address these questions we measured 22 mandibular cephalometric parameters that are measured routinely in most presurgical planning of human mandibular surgery and reconstruction. We then measured these on computed tomographic (CT) scans of Göttingen Minipigs aged between 12 and 21 months [45–48]. Ultimately, we compared our data with human data reported in the scientific literature. The parameters were chosen to evaluate the overall changes of the mandibular dimensions of subadult and adult Göttingen Minipigs. Measurements between the same landmarks on the left and right hemimandibles evaluated laterolateral growth, whilst distances between anterior and posterior landmarks served to evaluate longitudinal growth. Measurements between vertically located landmarks assessed the vertical growth of the mandibular ramus, whilst vertical parameters between the mental foramen and the alveolar ridge or the inferior border determined the posterior mental foramen’s vertical position. Manual segmentation of the coronoid and mandibular condyle was conducted to evaluate changes in their morphology and dimensions. In addition, image segmentation was used to determine the masticatory muscle morphology and the configuration of the mandibular blood vessels.

## Materials and methods

A computed tomographic study of Göttingen Minipigs approved by the Regional Office for Health and Social Affairs Berlin (permit IC113-G 0281/12) was conducted in 2007 and 2008 at the research facility for experimental surgery of the medical faculty (certified by ISO 9001) at Charité–Universitätsmedizin Berlin, Campus Virchow-Klinikum [49]. These CT scans were re-used for the cephalometric measurements of the present study. Whilst this precluded an optimal study design, it promoted the 3Rs by eliminating additional animal experiments.

### Animal groups and husbandry

The animals in this study consisted of 18 healthy female Göttingen Minipigs. Six animals were examined at the age of 12 months (12m; n = 6; 357 ± 31d) and another 12 animals were examined twice, once at 17 months (17m; n = 12; 511 ± 24d) and again at 21 months (21m; n = 11; 620 ± 37d). Their body mass ranged from 23 to 44 kg. Due to the loss of some of its data, one animal in the 21-month group was excluded from the study.

The minipigs were obtained from Ellegaard, Göttingen Minipigs (Dalmose, Denmark). To lessen the effects of humans as stressors, the animals had been habituated to routine handling and basic techniques such as blood sampling.

At the research facility in Berlin, the animals were housed according to the Guidelines of the European Societies of Laboratory Animal Science. The pigs were grouped into pens of six animals, with a relative humidity of 55 ± 10%, a light/dark rhythm of 12/12 hours and temperatures between 15 and 24°C. The animals were fed a specific diet formulated for minipigs to prevent obesity (Ssnif Spezialdiäten GmbH, Soest, Germany) [50]. Their body mass was measured weekly using a decimal scale.

### Adult human mandible

The image of a human mandible shown in the results, originated from a free anonymous CT-sample provided by the software company (Vital Images Inc., Minnetonka, MN, USA). The gender and exact age of the sample is unknown, however the overall mandibular dimensions indicate that it is from an adult person.

### Computed Tomography

#### Anaesthesia and drug administration

Prior to tomography, animals were fasted for 24 hours with water ad libitum. Premedication consisted of an intramuscular injection of 0.5 mg atropine (Atropinum sulfuricum, 1 mg/ml, Eifelfango, Bad Neuenahr-Ahrweiler, Germany). For the induction of anaesthesia, an intramuscular injection of ketamine (27 mg/kg, Ursotamin, 100 mg/ml, Serumwerk Bernburg, Germany), xylazine (3.5 mg/kg, Rompun TS, 20 mg/ml, Bayer Vital GmbH, Leverkusen, Germany) and 3 ml azaperone (Stresnil, 40 mg/ml, Janssen Animal Health, Neuss, Germany) was administered. Throughout the entire procedure, an isotonic electrolyte solution was infused intravenously (Ionosteril, Fresenius, Bad Homburg v. d. H., Germany) [51]. For separate studies on the vascular distribution of the whole body [49] and further histologic examination, all animals were euthanised when in deep anaesthesia by a 15 ml intravenous injection of T61 (Intervet Deutschland GmbH, Unterschleißheim, Germany).

#### Equipment and Software

The data acquisition was performed using a 64-slice scanner (Lightspeed 64, GE Medical Systems, Milwaukee, USA). For contrast enhancement, an automated intravenous injection of 80 ml nonionic iodinated contrast medium (XenetiX 350, Guerbet GmbH, Sulzbach, Germany 350 mg iodine /ml) was used in every pig. Scanning parameters were standardised (voltage of 120 kV, an amperage of 500 mA with automatic mA-optimization at a noise index of 15, mean 490 mA; collimated slice thickness of 64×0.625 mm, total detector width of 55 mm, rotation speed of 0.4 sec and table feed per rotation of 55 mm) [52]. The positioning and the following computed tomographic examination required only a few minutes per animal. The 12m minipigs were imaged twice over 27 days, and the 17m and 21m minipigs were imaged five times over 111 days. Then the data was transferred to an independent workstation and the software Vitrea Advanced 6.6 (Vital Images Inc., Minnetonka, MN, USA) was used for measurements, segmentation and 3D rendering. Without overlap of images, the volumetric assessment was reconstructed with a slice thickness of 1.25 mm.

### Anatomical landmarks

The definitions of the cephalometric landmarks used in this study are presented in Table 1. These landmarks are derived primarily from anthropometric landmarks that have been defined and modified by different authors over many decades [53, 54].

**Table 1:** Cephalometric landmarks and their definitions. List of the anatomical landmarks that were used in this study and their definition, listed in anterior to posterior order.

Landmark	Definition
Infradentale (Id)	The apex of the septum between the mandibular central incisors [55].
Menton (Me)	Lowest midsagittal point of the intermandibular symphysis [55].
Diastema (Dia)	Prominent toothless gap of each hemimandible, located between the canine and the premolar teeth.
Midpoint of the diastema (mDia)	Midtransversal point of the diastema.
Mental foramen (Mf)	Posterior prominent mental foramen.
Alveolar crest (Ac)	Point on the buccal alveolar crest at the level of the posterior mental foramen (Mf).
Inferior border (Ib)	Most inferior point of the mandibular body at the level of the posterior mental foramen (Mf).
Dental ridge length (Ld)	Length of the premolar and molar dental arch.
Coronion (Cor)	Most superior point of the coronoid process.
Condylion (Con)	Most superior point of the mandibular condyle.
Lowest point of the sigmoid notch (Sn)	Most inferior point of the sigmoid notch, located between the coronoid and mandibular process.
Gonion (Go)	Most posterior, inferior and lateral point on the external angle of the mandible [56].

### Parameters measured

**Fig 1:**
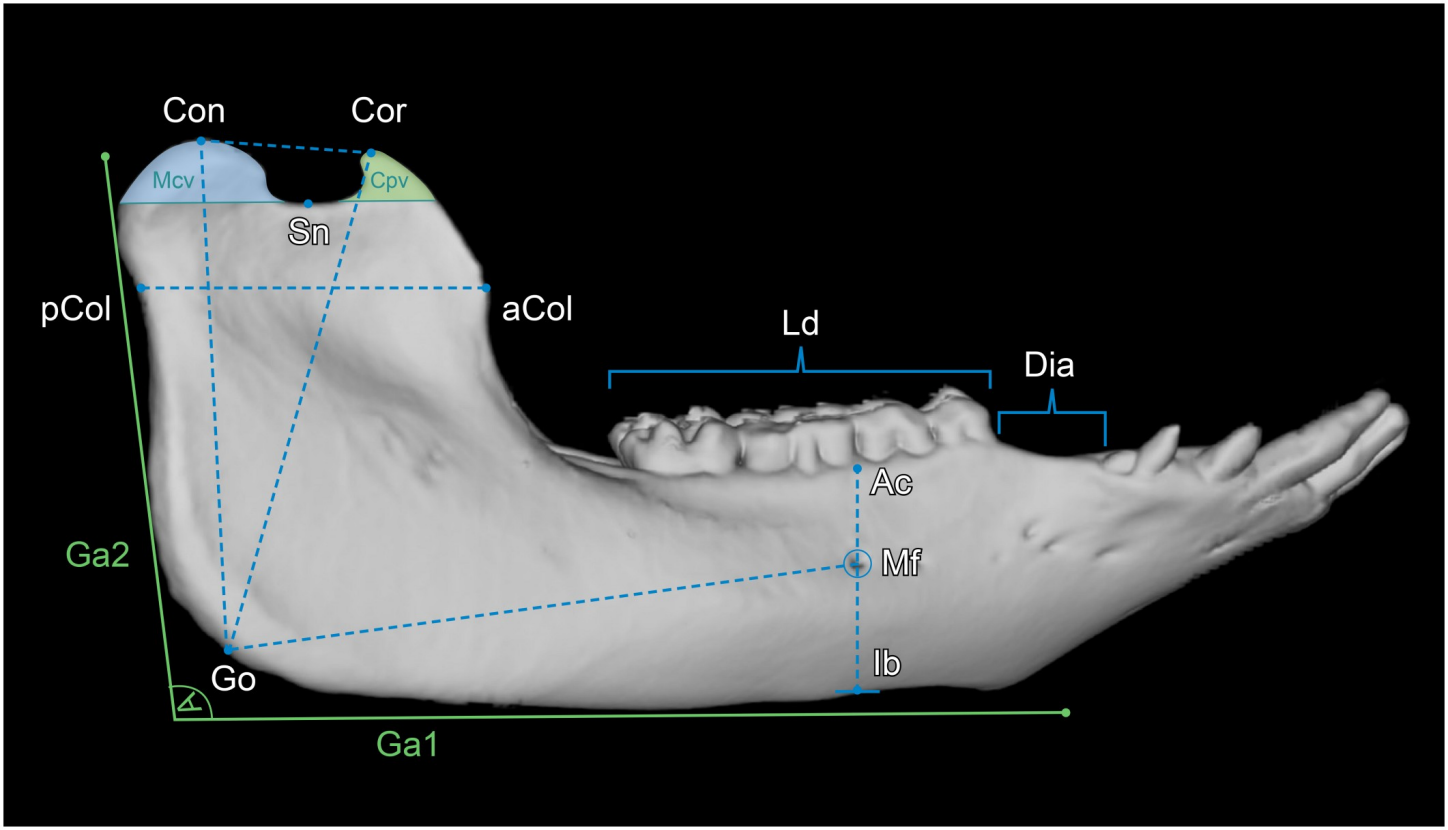
Lateral view of a 3D rendered mandible of a 17 months-old Göttingen Minipig with landmarks and measured parameters. Where: Con = condylion, Cor = coronion, Sn = lowest point of the sigmoid notch, pCol = posterior point of the mandibular collum, aCol = anterior point of the mandibular collum, Ga1 = horizontal tangent alongside the inferior border of the mandibular body, Ga2 = near vertical tangent alongside the posterior border of the mandibular ramus, Ac = point on the buccal alveolar crest at the vertical level of the posterior mental foramen, Mf = posterior prominent mental foramen, Ib = most inferior point of the mandibular body at the vertical level of the posterior mental foramen, Go = gonion. The parameters measured were: Con–Go = mandibular ramus height (MRH), Cor–Go = oblique mandibular ramus height (oMRH), Dia = diastemal length (DL), Ld = premolar and molar dental arch length (DAL), Mf–Ib = mental foramen to inferior border (MIB), Mf–Ac = mental foramen to alveolar crest (MAC), Mf–Go = mental foramen to gonion (MGO), Ga1-Ga2 = gonial angle (GA), aCol–pCol = mandibular ramus length (MRL), Cor–Con = superior ramus length (SRL), Cpv = coronoid process volume (CPV), Mcv = mandibular condyle volume (MCV).

**Fig 2:**
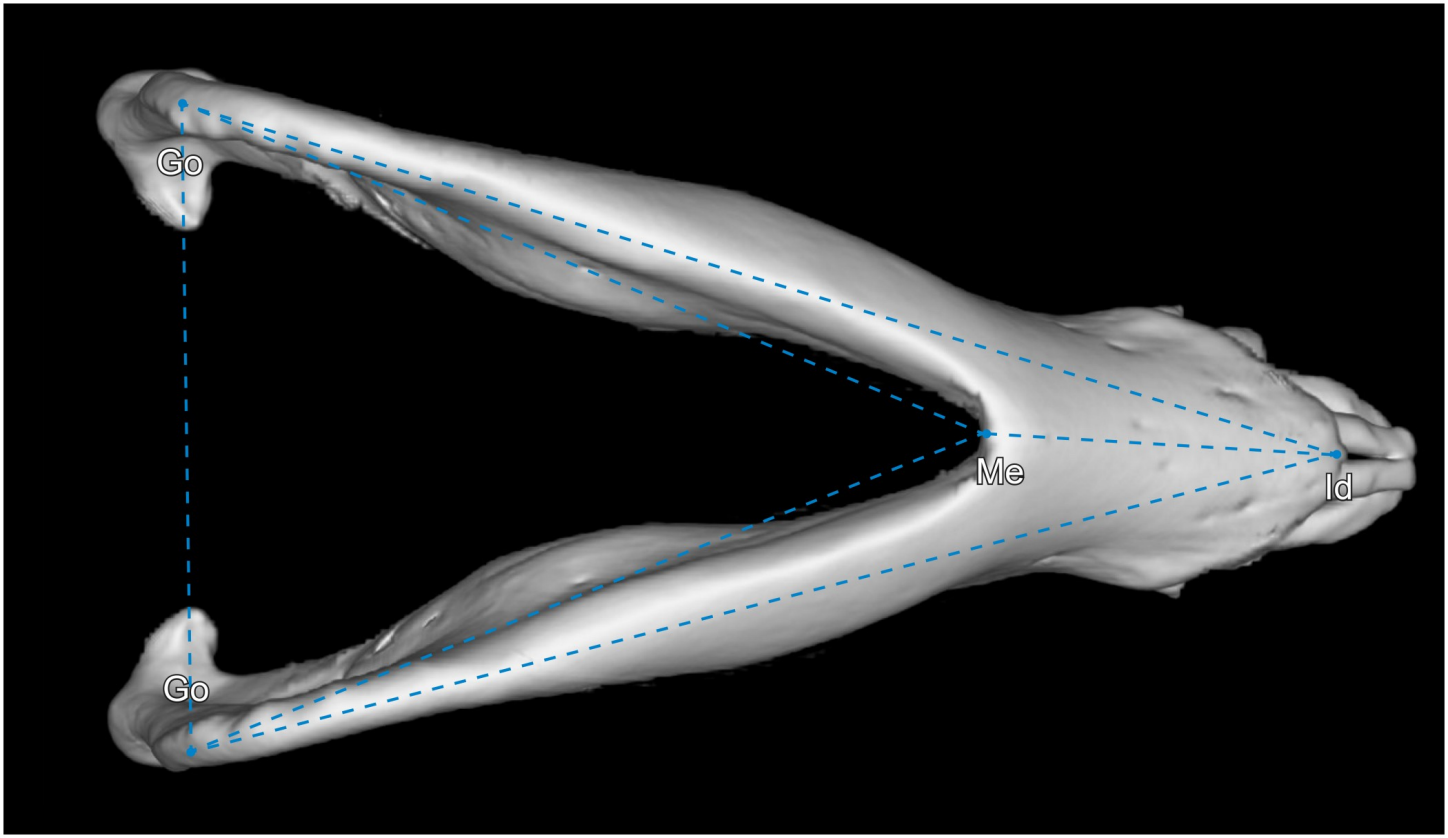
Inferior view of a 3D rendered mandible of a 17 months-old Göttingen Minipig with landmarks and measured parameters. Where: Go = gonion, Me = menton, Id = infradentale. The parameters measured were: Go–Go = intergonial breadth (IGB), Go–Me = inferior mandibular body length (iMBL), Go–Id = mandibular body length (MBL), Me–Id = anterior mentum height (AMH).

**Fig 3:**
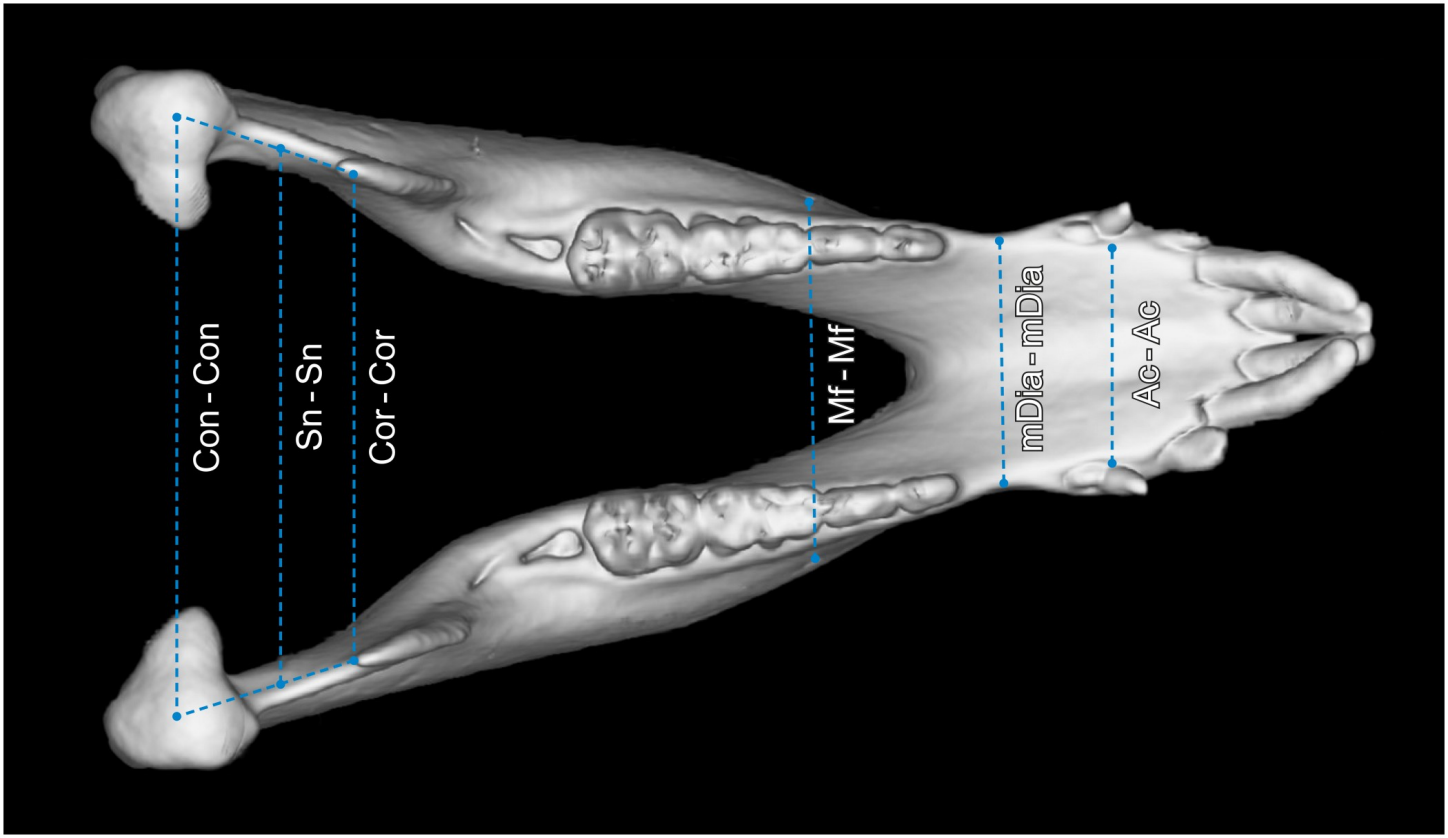
Superior view of a 3D rendered mandible of a 17 months-old Göttingen Minipig with landmarks and measured parameters. Where: Con = condylion, Cor = coronion, Sn = lowest point of the sigmoid notch, Mf = posterior prominent mental foramen, mDia = midpoint of the diastema, Ac = point on the buccal alveolar crest at the vertical level of the posterior mental foramen. The parameters measured were: mDia–mDia = interdiastemal breadth (IB), Ac–Ac = lingual intercrestal breadth (LIB), Mf–Mf = interforaminal breadth (IFB), Cor–Cor = intercoronoidal breadth (ICOB), Sn–Sn = breadth between sigmoid notches (SNB) and Con–Con = intercondylar breadth (ICB).

Except for the coronoid process volume (CPV) and the mandibular condyle volume (MCV), all parameters measured are distances between two defined landmarks (Table 1). For the segmentation and calculation of CPV and MVC, as well as for the segmentation of the mandibular condyles, the masticatory muscles and the whole mandible, the “sculpt” function of Vitrea Advanced was used. To evaluate the different morphologies of mandibles of humans and minipigs, two segmentations were scaled to the same size and superimposed upon each other. To ensure high reproducibility and for the correct identification of landmarks, multiplanar (sagittal, coronal, axial) views that were automatically reconstructed from the original axial slices, were used. In addition, bone reconstruction kernels were applied (Bone plus, GE Medical Systems, Milwaukee, USA) [17]. Table 2 lists all measured parameters, their abbreviations and definitions. All parameters were measured on both left and right hemimandibles. All parameters are given in millimeters (mm) except for CPV, MCV and GA that are given in cubic millimeters (mm^3^), millilitres (ml) and degrees. In Figs 1–3, a segmented mandible of a 17 months-old Göttingen Minipigs is pictured with all landmarks and measured parameters.

**Table 2:** List of the cephalometric parameters, their abbreviations and definitions. Parameters are described by distances between two distinct anatomical landmarks, which are defined in Table 1.

Abbreviation	Parameters	Definition	Figure
**MRH**	Mandibular ramus height	Con—Go	1
**oMRH**	Oblique mandibular ramus height	Cor—Go	1
**iMBL**	Inferior mandibular body length	Go—Me	2
**MBL**	Mandibular body length	Go—Id	2
**DL**	Diastemal length	Dia	1
**DAL**	Premolar and molar dental arch length	Ld	1
**IB**	Interdiastemal breadth	mDia—mDia	3
**LIB**	Lingual intercrestal breadth	Ac—Ac	3
**MIB**	Mental foramen to inferior mandibular border height	Mf—Ib	1
**MAC**	Mental foramen to alveolar crest height	Mf—Ac	1
**MGO**	Mental foramen to gonion length	Mf—Go	1
**IFB**	Interforaminal breadth	Mf—Mf	3
**GA**	Gonial angle	Ga	1
**MRL**	Mandibular ramus length	aCol—pCol	1
**SRL**	Superior ramus length	Cor—Con	1
**CPV**	Coronoid process volume	Cpv	1
**MCV**	Mandibular condyle volume	Mcv	1
**AMH**	Anterior mentum height	Me—Id	2
**ICOB**	Intercoronoidal breadth	Cor—Cor	3
**SNB**	Breadth between sigmoid notches	Sn—Sn	3
**ICB**	Intercondylar breadth	Con—Con	3
**IGB**	Intergonial breadth	Go—Go	2

The diastemal length (DL) is the length of the toothless gap from the distal aspect of the canine tooth to the mesial aspect of the premolar tooth (Fig 1).

The premolar and molar dental arch length (DAL) was measured from the mesial aspect of the premolar to the posterior surface of the last molar tooth (Fig 1).

The interdiastemal breadth (IB) is the distance between both midpoints (mDia) of the diastemal length (Fig 3).

The lingual intercrestal breadth (LIB) is the distance connecting the lingual alveolar crests (Ac) of the canine teeth (Fig 3).

The parameters MIB, MAC and MGO describe the position of the mental foramen (Mf).

The gonial angle (GA) is the angle measured between two intersecting tangents. Tangent 1 runs horizontally alongside the inferior border of the mandibular body (Ga1), and tangent 2 runs vertically alongside the posterior border of the mandibular ramus (Ga2) (Fig 1).

The lines for calculating the coronoid process volume (CPV) and the mandibular condyle volume (MCV) were drawn manually in the coronal plane, starting from the coronion and the condylion to a horizontal plane through the inferior point of the sigmoid notch (Sn) (Fig 1).

### Statistics

IBM SPSS Statistics 23 was used for statistical analysis (IBM Deutschland GmbH, Kassel, Germany). All parameters were checked for normal distribution. If normal distribution was revealed, the student’s *t*-Test was used. For non-normal distributed data, the Mann-Whitney-U-, Wilcoxon- and Kruskall-Wallis Tests were utilised. For the comparison of 12m animals with animals of 17m and 21m, the Independent T-test was used because the animals in the 12m group differ from those of 17m and 21m. Because the animals in 17m and 21m groups were the same individuals measured at different time points, they were treated statistically as paired samples and the Paired-student’s *t*-Test was used. Correlations between parameters were analyzed with the bivariate Pearson-Test or Spearman-Rho-Test, depending whether normal or non-normal distributed data was present. Values are given as mean values with the associated standard deviations. A *p* value of less than 0.05 was considered significant. Correlation coefficients (r) between 0.45 to 0.59 were considered to be moderate correlations, whereas correlation coefficients between 0.60 to 0.79 were considered as strong and from 0.80 to 1.0 to be very strong correlations. All measurements were executed by the same trained examiner (GMC) and under the supervision of an experienced radiologist (SMN). For the estimation of the observer’s reproducibility of the measured values, several blind tests were conducted and it was proven that the measurements were precise and reliable.

#### Comparison with human data from literature

The relationship of age specific values of minipigs and human data, averaged over all available published means, was expressed as a minipig-human ratio (MP:H). Ratios lower than 0.85 and higher than 1.15 were defined as substantial anatomical deviations between both species. Parameters with ratios within the range of 0.85 and 1.15 were considered to have a moderate (>0.85 and <1.15) or high (>0.9 and <1.1) comparability.

## Results

The mean values, standard deviations and p-values of all parameters measured are presented in Table 3. The p-values are the results of the statistical hypothesis tests conducted to determine if the parameter data of the three minipig age groups differ significantly from each other. Depending whether normal or non-normal distribution was present, student’s t- (Independent and Paired), Mann-Whitney-U-, Wilcoxon- or Kruskall-Wallis Test was utilized. The data of left and right hemimandibles did not show any significant differences and were therefore pooled. All parameters (Table 3) showed significant correlations between the left and right hemimandibles and therefore no significant asymmetries were observable.

**Table 3:** Mean values, standard deviations and p-values of all measured parameters. The data of left and right hemimandibles were statistically similar and were therefore pooled. The p-values presented are the results of the statistical hypothesis tests conducted to determine if the parameter data of the three minipig age groups differ significantly from each other.

Parameter	12 months (n = 6)	17 months (n = 12)	21 months (n = 11)	p-values
				1) 12m-17m
				2) 12m-21m
				3) 17m-21m
**Mandibular ramus height [mm] (MRH)**	73.43 ± 3.44	78.08 ± 3.88	81.58 ± 4.00	0.001
				0.000
				0.012
**Oblique mandibular ramus height [mm] (oMRH)**	74.51 ± 3.69	77.15 ± 3.58	81.46 ± 4.98	0.047
				0.015
				0.026
**Inferior mandibular body length [mm] (iMBL)**	105.70 ± 2.68	112.49 ± 3.32	120.33 ± 2.90	0.000
				0.000
				0.000
**Mandibular body length [mm] (MBL)**	144.30 ± 5.23	152.14 ± 3.19	160.40 ± 4.06	0.000
				0.000
				0.000
**Diastemal length [mm] (DL)**	14.57 ± 2.40	14.54 ± 1.47	15.30 ± 1.70	0.970
				0.316
				0.245
**Premolar and molar dental arch length [mm] (DAL)**	57.19 ± 7.81	61.27 ± 1.60	63.15 ± 5.62	0.753
				0.444
				0.807
**Interdiastemal breadth [mm] (IB)**	33.38 ± 1.42	35.48 ± 1.28	37.57 ± 1.24	0.006
				0.000
				0.001
**Lingual intercrestal breadth [mm] (LIB)**	28.30 ± 1.24	29.19 ± 1.76	30.37 ± 2.06	0.289
				0.041
				0.152
**Mental foramen to inferior mandible border height [mm] (MIB)**	20.59 ± 1.62	22.90 ± 2.08	23.87 ± 2.12	0.002
				0.000
				0.144
**Mental foramen to alveolar crest height [mm] (MAC)**	13.40 ± 1.82	10.62 ± 1.50	11.40 ± 1.63	0.000
				0.003
				0.140
**Mental foramen to gonion length [mm] (MGO)**	81.57 ± 2.56	85.71 ± 3.15	90.92 ± 3.70	0.000
				0.000
				0.000
**Interforaminal breadth [mm] (IFB)**	51.59 ± 2.93	55.79 ± 2.86	57.91 ± 2.75	0.010
				0.000
				0.108
**Gonial angle [degree] (GA)**	97.53 ± 4.43	99.36 ± 3.80	97.32 ± 4.42	0.205
				0.898
				0.118
**Mandibular ramus length [mm] (MRL)**	42.27 ± 2.12	42.27 ± 1.48	44.46 ± 1.13	0.998
				0.001
				0.000
**Superior ramus length [mm] (SRL)**	25.42 ± 2.22	27.42 ± 1.56	27.43 ± 1.73	0.004
				0.006
				0.988
**Coronoid process volume [mm**^**3**^**] (CPV)**	193.42 ± 82.29	108.19 ± 56.39	187.75 ± 95.02	0.009
				0.719
				0.016
**Mandibular condyle volume [ml] (MCV)**	1.94 ± 0.52	2.68 ± 0.39	2.94 ± 0.65	0.000
				0.000
				0.045
**Anterior mentum height [mm] (AMH)**	45.92 ± 4.46	46.00 ± 2.76	48.03 ± 3.36	0.963
				0.286
				0.066
**Intercoronoidal breadth [mm] (ICOB)**	68.01 ± 2.36	71.44 ± 1.75	73.51 ± 1.59	0.003
				0.000
				0.015
**Breadth between sigmoid notches [mm] (SNB)**	75.29 ± 4.27	77.44 ± 3.66	80.06 ± 2.86	0.283
				0.014
				0.107
**Intercondylar breadth [mm] (ICB)**	88.08 ± 4.08	88.84 ± 2.85	91.61 ± 3.54	0.648
				0.082
				0.052
**Intergonial breadth [mm] (IGB)**	96.95 ± 7.72	106.62 ± 3.58	111.83 ± 4.79	0.002
				0.000
				0.002

**Fig 4:**
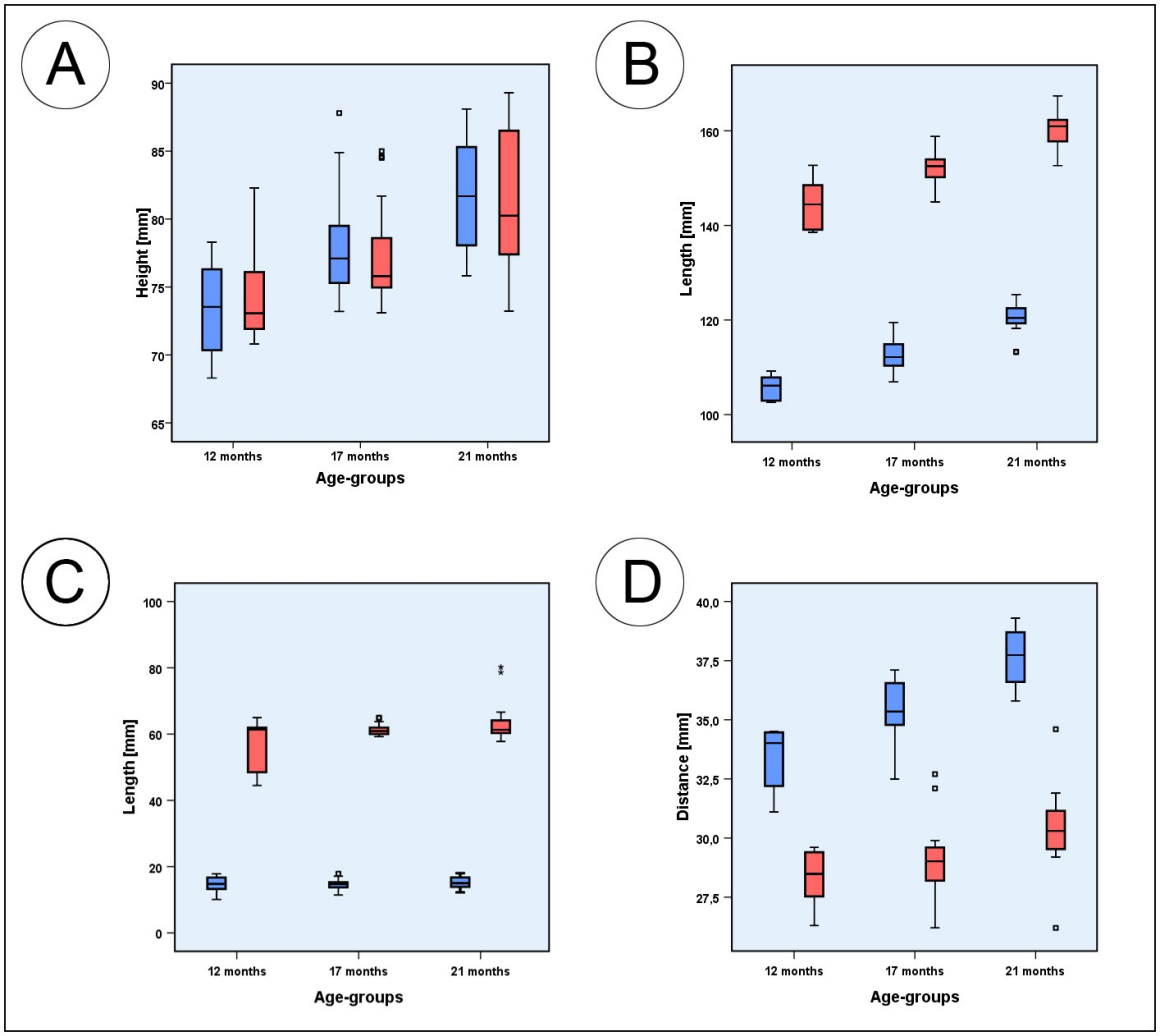
Box plots of measured parameters. (A) Box plots of the mandibular ramus height (blue) and oblique mandibular ramus height (red); (B) Box plots of the inferior mandibular body length (blue) and mandibular body length (red); (C) Box plots of the diastemal length (blue) and the premolar and molar dental arch length (red); (D) Box plots of the interdiastemal breadth (blue) and lingual intercrestal breadth (red). Squares associated with the box plots are individual outliers. Outliers marked with asterisks are values that exceed the triple interquartile range.

**Fig 5:**
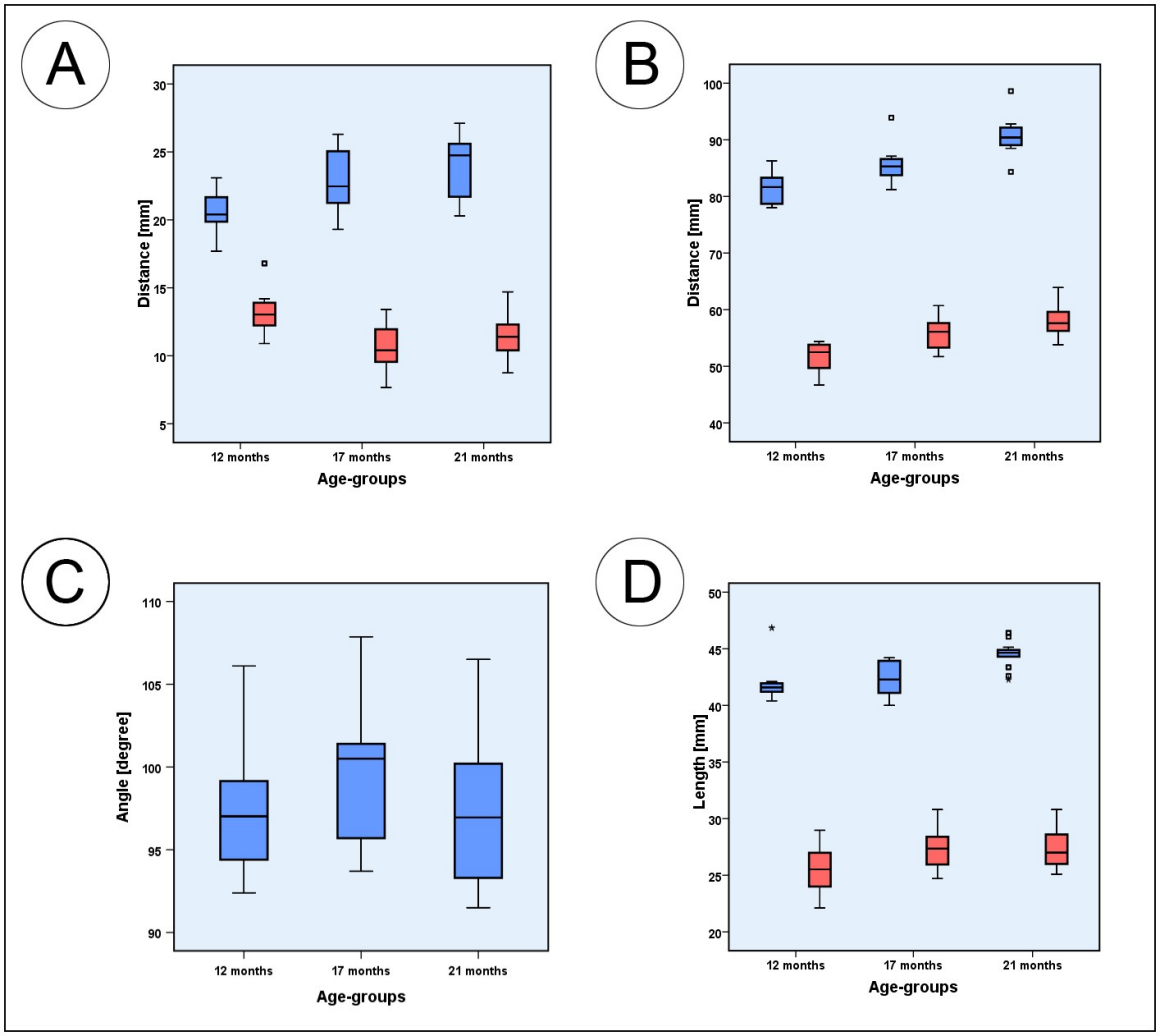
Box plots of measured parameters. (A) Box plots of mental foramen to inferior mandible border height (blue), and mental foramen to mandibular alveolar crest height (red); (B) mental foramen to gonion length (blue) and interforaminal breadth (red). (C) Box plots of the gonial angle measurements (blue); (D) Box plots of the mandibular ramus length (blue) and superior ramus length (red). Squares associated with the box plots are individual outliers. Outliers that are marked with asterisks are values that exceed the triple interquartile range.

**Fig 6:**
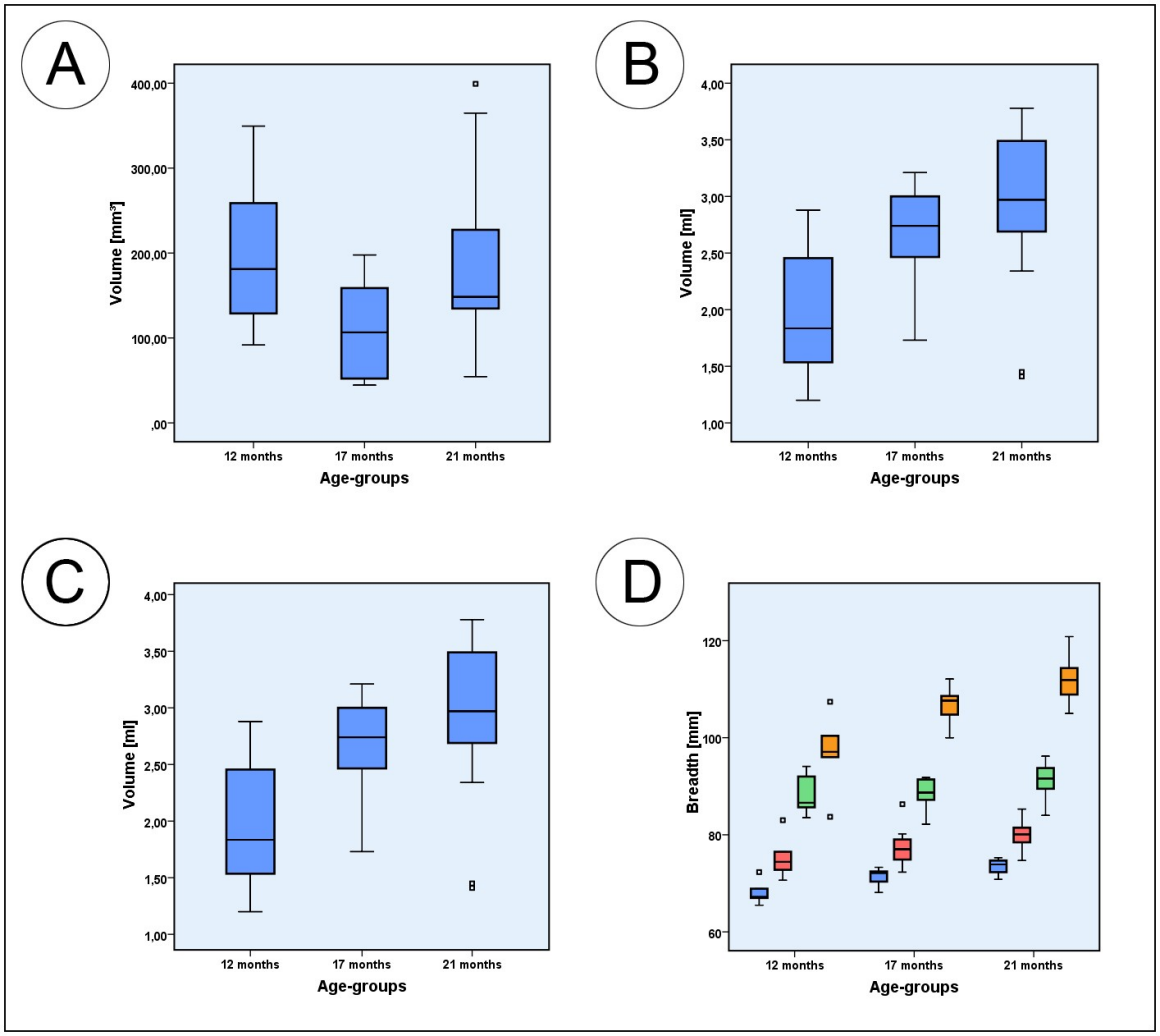
Box plots of measured parameters. (A) Box plots of the coronoid process volume; (B) Box plots of the mandibular condyle volume; (C) Box plots of the anterior mentum height; (D) Box plots of the intercoronoidal breadth (blue); intercondylar breadth (green); intergonial breadth (orange) and breath between sigmoid notches (red). Squares associated with the box plots are individual outliers.

Table 4 presents an overview of all parameters measured indicating significant changes, lowest and highest individual values as well as correlations between the left and right hemimandible, with age and with body mass. The Figs 4–6 are boxplots of all measured parameters.

**Table 4:** Overview of significant changes, lowest and highest individual values and correlations. Significant correlations are pictured in green, negative correlation in yellow and non-significant values in red. Correlations were considered moderate (0.45 to 0.59), strong (0.60 to 0.79) and very strong (0.80 to 1.0). Significance levels are reported as *p<0.05, **p<0.01, ***p<0.001.

Param.	Significant changes with age	Lowest individual value (Group)	Highest individual value (Group)	Correlation between left and right hemi-mandible	Correlation with age	Correlation with body mass
**MRH [mm]**	Increase	68.3 (12m)	88.1 (21m)	r = 0.977***	r = 0.685***	r = 0.508***
**oMRH [mm]**	Increase	70.8 (12m)	89.3 (21m)	r = 0.974***	r = 0.686***	r = 0.327
**iMBL [mm]**	Increase	102.6 (12m)	125.4 (21m)	r = 0.968***	r = 0.835***	r = 0.671***
**MBL [mm]**	Increase	138.6 (12m)	167.4 (21m)	r = 0.959***	r = 0.832***	r = 0.511***
**DL [mm]**	No changes	10.1 (12m)	18.1 (21m)	r = 0.719***	r = 0.132	r = 0.103
**DAL [mm]**	No changes	44.5 (12m)	80.2 (21m)	r = 0.481***	r = 0.188	r = 0.059
**IB [mm]**	Increase	31.1 (12m)	39.3 (21m)	---	r = 0.799***	r = 0.487**
**LIB [mm]**	Increase when comparing 12 and 21m	26.2 (17m)	34.6 (21m)	---	r = 0.440**	r = 0.041
**MIB [mm]**	Increases between 12 and 17 m. No change after 17m	17.7 (12m)	27.1 (21m)	r = 0.918***	r = 0.490***	r = 0.110
**MAC [mm]**	Decreases between 12 and 17m. No change after 17m.	7.7 (17m)	16.8 (12m)	r = 0.593***	r = -0.194	r = -0.209
**MGO [mm]**	Increase	78.0 (12m)	99.2 (21m)	r = 0.932***	r = 0.782***	r = 0.610***
**IFB [mm]**	Increases between 12 and 17 m. No change after 17m	46.7 (12m)	63.9 (21m)	---	r = 0.563***	r = 0.080
**GA [degree]**	No change	91.5° (21m)	107.9° (17m)	r = 0.951***	r = -0.130	r = -0.367**
**MRL [mm]**	Change after 17m.	40.0 (17m)	46.9 (12m)	r = 0.946***	r = 0.373***	r = 0.392***
**SRL [mm]**	Increase between 12 and 17 m. No change after 17m.	22. (12m)	30.8 (21m)	r = 0.932***	r = 0.348**	r = 0.448***
**CPV [mm3]**	Only when directly comparing 12-17m and 17-21m	44.5 (17m)	399.2 (21m)	r = 0.958***	r = 0.013	r = -0.130
**MCV [ml]**	Increase	1.2 (12m)	3.8 (21m)	r = 0.907***	r = 0.581**	r = 0.623**
**AMH [mm]**	No change	41.7 (12m)	54.7 (21m)	---	r = 0.220	r = 0.135
**ICOB [mm]**	Increase	65.5 (12m)	75.3 (21m)	---	r = 0.761***	r = 0.451*
**SNB [mm]**	Significant when directly comparing 12m and 21m	70.6 (12m)	86.3 (21m)	---	r = 0.473**	r = 0.137
**ICB [mm]**	No change	82.2 (17m)	96.2 (21m)	---	r = 0.349	r = 0.638***
**IGB [mm]**	Increase	83.7 (12m)	120.8 (21m)	---	r = 0.781***	r = 0.621***

### Comparison to human data

The comparison to human data (Table 5) shows that 4 parameters, namely the MAC, MGO, MCV and IGB are highly comparable between the two species. Three other parameters have moderate comparability. All others are either not comparable or could not be compared due to insufficient data in the literature.

**Table 5:** Comparison to human data. The total mean values of all parameters measured in each of the three minipig age groups and the corresponding data of humans from the scientific literature. The three different colors identify the differences between the age-group values of the minipigs and the mean values of human data, presented as a minipig/human ratio (MP:H). Ratios lower than 0.85 and higher than 1.15 were defined as substantial anatomical deviations (in red) between the two species where no comparability is present. Parameters with ratios within the range of 0.85 and 1.15 were considered to have a moderate (>0.85 and <1.15 in yellow) or a high (>0.9 and <1.1 in green) comparability.

Param.	Age (m)	Minipigs ($\bar{\text{x}}$ ± SD)	Humans ($\bar{\text{x}}$ ± SD)	Authors	MP/H-ratio
**MRH**	12	73.4 ± 3.4 mm	53.1 ± 5.3 mm; 56.5 ± 5.1 mm; 57.6 ± 5.8 mm; 59.3 mm	Lopez et al. [57]; Moshiri et al. [58]; Bayome et al. [59]; Ozturk et al. [60]Moshiri et al. [58]Ozturk et al. [60]	0.771
	17	78.1 ± 3.9 mm			0.725
	21	81.6 ± 4.0 mm			0.694
**MRH**	12	74.5 ± 3.7 mm	53.2 ± 3.6 mm; 64.5 ± 4.2 mm	Franklin et al. [61]; Kim et al. [62]	0.789
	17	77.2 ± 3.6 mm			0.762
	21	81.5 ± 5.0 mm			0.721
**iMBL**	12	105.7 ± 2.7 mm	72.7 ± 5.3 mm; 79.4 ± 5.6 mm; 79.4 ± 5.6 mm; 88.0 ± 5.0 mm and 93.0 ± 5.0 mm	Steyn and Iscan [63]; Moshiri et al. [58]; Bayome et al. [59]; Weijs and Hillen [64]	0.787
	17	112.5 ± 3.3 mm			0.740
	21	120.3 ± 2.9 mm			0.692
**MBL**	12	144.3 ± 5.2 mm	No comparison possible		
	17	152.1 ± 3.2 mm			
	21	160.4 ± 4.1 mm			
**DL**	12	14.6 ± 2.4 mm	No comparison possible		
	17	14.5 ± 1.5 mm			
	21	15.3 ± 1.7 mm			
**DAL**	12	57.2 ± 7.8 mm	38.4 ± 2.7 mm; 41.5 and 44.7 mm	Al-Zubair et al. [65]; Braun et al. [66]	0.726
	17	61.3 ± 1.6 mm			0.678
	21	63.2 ± 5.6 mm			0.657
**IB**	12	33.4 ± 1.4 mm	No comparison possible		
	17	35.5 ± 1.3 mm			
	21	37.6 ± 1.2 mm			
**LIB**	12	28.3 ± 1.2 mm	24.4 ± 1.4 mm; 25.4 ± 1.8 mm; 25.3 ± 0.9 mm and 26.4 ± 2.9 mm	Bishara et al. [67]; Singh et al. [68]; Tamewar et al. [69]	0.897
	17	29.2 ± 1.8 mm			0.869
	21	30.4 ± 2.1 mm			0.802
**MIB**	12	20.6 ± 1.6 mm	11.5 mm; 15.2 mm	Ozturk et al. [60]; Tebo and Telford [70]	0.648
	17	22.9 ± 2.1 mm			0.583
	21	23.9 ± 2.1 mm			0.559
**MAC**	12	13.4 ± 1.8 mm	11.4 mm; 11.8 ± 3.0 mm	Ozturk et al. [60]; Lorenzo et al. [71]	0.866
	17	10.6 ± 1.5 mm			0.906
	21	11.4 ± 1.6 mm			1.017
**MGO**	12	81.6 ± 2.6 mm	74.6 mm	Tebo and Telford [70]	0.914
	17	85.7 ± 3.2 mm			0.870
	21	90.9 ± 3.7 mm			0.820
**IFB**	12	51.6 ± 2.9 mm	44.6 ± 2.5 mm; 43.2 ± 2.8 mm; 47.2 ± 2.8 mm and 49.9 ± 3.0 mm	Lopez et al. [57]; Kumar et al. [72]; Dong et al. [73]	0.853
	17	55.8 ± 2.9 mm			0.806
	21	57.9 ± 2.8 mm			0.781
**GA**	12	97.5 ± 4.4°	115.5 ± 4.0°; 118.6 ± 5.2°; 123.9 ± 7.3°; 125.7 ± 5.6°	Bayome et al. [59]; Weijs and Hillen [64]; Lopez et al. [57]; Dong et al. [73]	1.194
	17	99.4 ± 3.8°			1.178
	21	97.3 ± 4.4°			1.195
**MRL**	12	42.3 ± 2.1 mm	32.7 ± 2.8 mm; 37.8 ± 2.9 mm and 39.8 ± 3.7 mm	Kim et al. [62]; Giles [74]	0.869
	17	42.3 ± 1.5 mm			0.869
	21	44.5 ± 1.1 mm			0.826
**SRL**	12	25.4 ± 2.2 mm	31.3 ± 2.9 mm; 33.5 ± 3.6 mm	Lopez et al. [57]; Kim et al. [75]	1.216
	17	27.4 ± 1.6 mm			1.154
	21	27.4 ± 1.7 mm			1.154
**CPV**	12	193.4 ± 82.3 mm³	250.0 ± 9.0 mm³	Gomes et al. [76]	1.226
	17	108.2 ± 56.4 mm³			1.567
	21	187.8 ± 95.0 mm³			1.249
**MCV**	12	1.9 ± 0.5 ml	2.3 ml and 2.4 ml; 2.7 ± 0.4 ml	Safi et al. [77]; Saccucci et al. [78]	1.230
	17	2.7 ± 0.4 ml			0.914
	21	2.9 ± 0.7 ml			0.851
**AMH**	12	45.9 ± 4.5 mm	24.6 mm; 28.5 ± 3.0 mm; 29.6 ± 3.5 mm	Ozturk et al. [60]; Giles [74]; Kumar et al. [72]	0.600
	17	46.0 ± 2.8 mm			0.599
	21	48.0 ± 3.4 mm			0.574
**ICOB**	12	68.0 ± 2.4 mm	90.8 ± 5.7 mm; 92.0 ± 5.7 mm	Lopez et al. [57]; Kumar et al. [72]	1.251
	17	71.4 ± 1.8 mm			1.219
	21	73.5 ± 1.6 mm			1.244
**SNB**	12	75.3 ± 4.3 mm	No available data		
	17	77.4 ± 3.7 mm			
	21	80.1 ± 2.9 mm			
**ICB**	12	88.1 ± 4.1 mm	111.2 ± 6.2 mm and 117.0 ± 5.3 mm; 110.5 ± 6.2 mm and 116.4 ± 7.0 mm	Steyn and Iscan [63]; Lopez et al. [57]	1.226
	17	88.9 ± 2.9 mm			1.219
	21	91.6 ± 3.5 mm			1.195
**IGB**	12	97.0 ± 7.7 mm	85.9 ± 5.0 mm; 91.5 ± 5.0 mm; 91.8 ± 5.9 mm; 93.7 ± 6.8 mm	Ozturk et al. [60]; Steyn and Iscan [63]; Lopez et al. [57]; Carvalho et al. [79]	0.935
	17	106.6 ± 3.6 mm			0.851
	21	111.8 ± 4.8 mm			0.811

### Visualization of the growth changes

**Fig 7:**
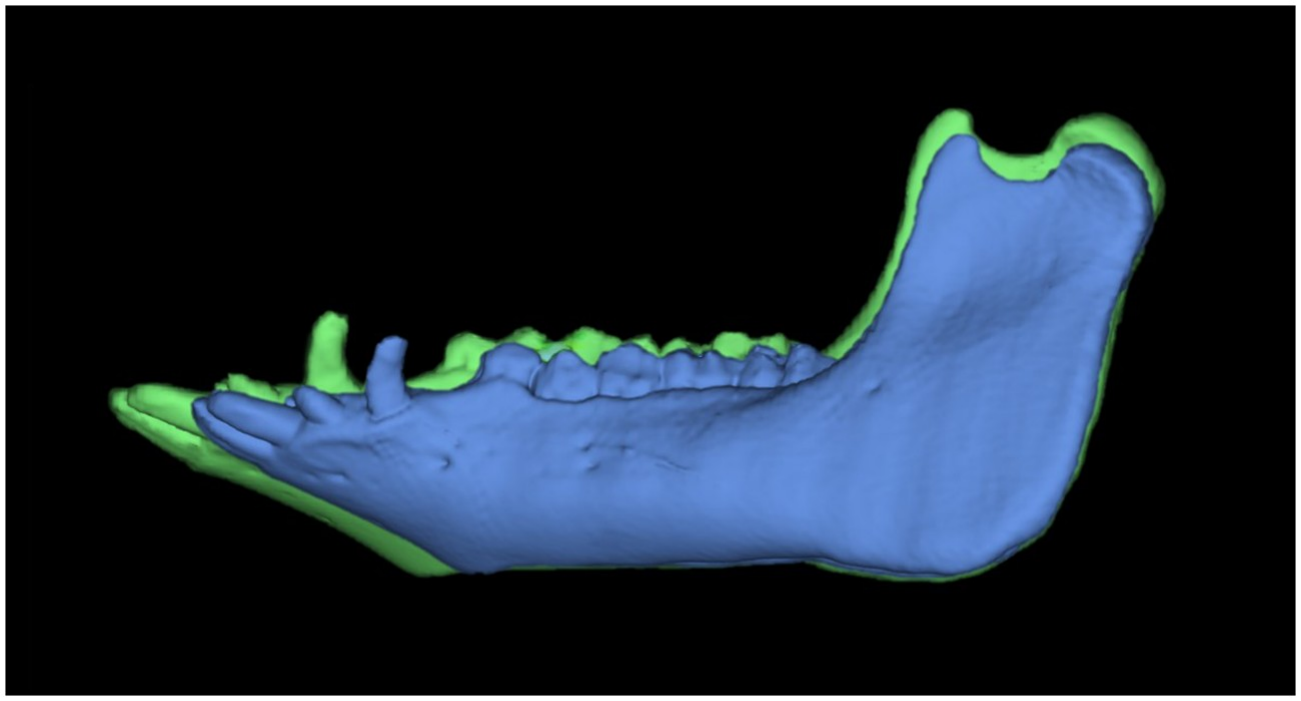
Lateral view of the same segmented mandible showing growth changes. The segmentations show the mandibular volume at 17 (blue) and 21 (green) months of age, merged and presented at the same scale.

Between 17 and 21 months of age, there is an obvious increase in mandibular body length, mandibular ramus height and oblique mandibular ramus height. The gonial angle does not change visually (Fig 7).

**Fig 8:**
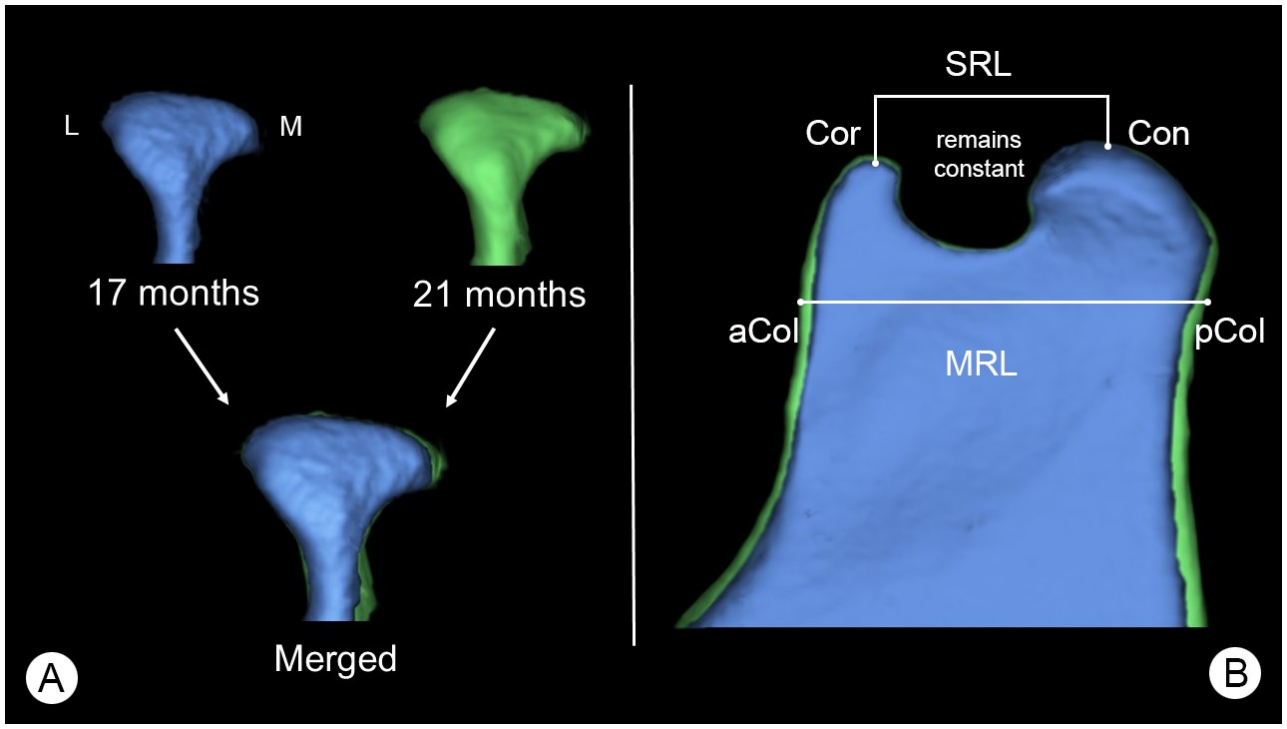
Growth changes of the mandibular condyle and superior ramus. (A) Posterior view of a mandibular condyle of the same individual animal at 17 (blue) and 21 months (green) of age scaled to same size, showing changes in mandibular condyle volume (MCV) over time. Here: L = lateral aspect of the mandibular condyle and M = medial aspect of the mandibular condyle. (B) Lateral view of the superior area of the mandibular ramus, showing growth changes of the mandibular ramus. Here: Cor = coronion, Con = condylion, aCol = anterior point of the mandibular collum, pCol = posterior point of the mandibular collum. Parameters were: SRL = Cor-Con, MRL = aCol-pCol. Segmentations are presented at the same scale.

Between 17 and 21 months, the mandibular condyle (Fig 8A) has an increase in horizontal width, with greater growth at its medial aspect. Beneath the condyle, the upper mandibular ramus increases in thickness over time. In addition, there is a slight increase in mandibular ramus length (Fig 8B). The superior mandibular ramus length does not change.

**Fig 9:**
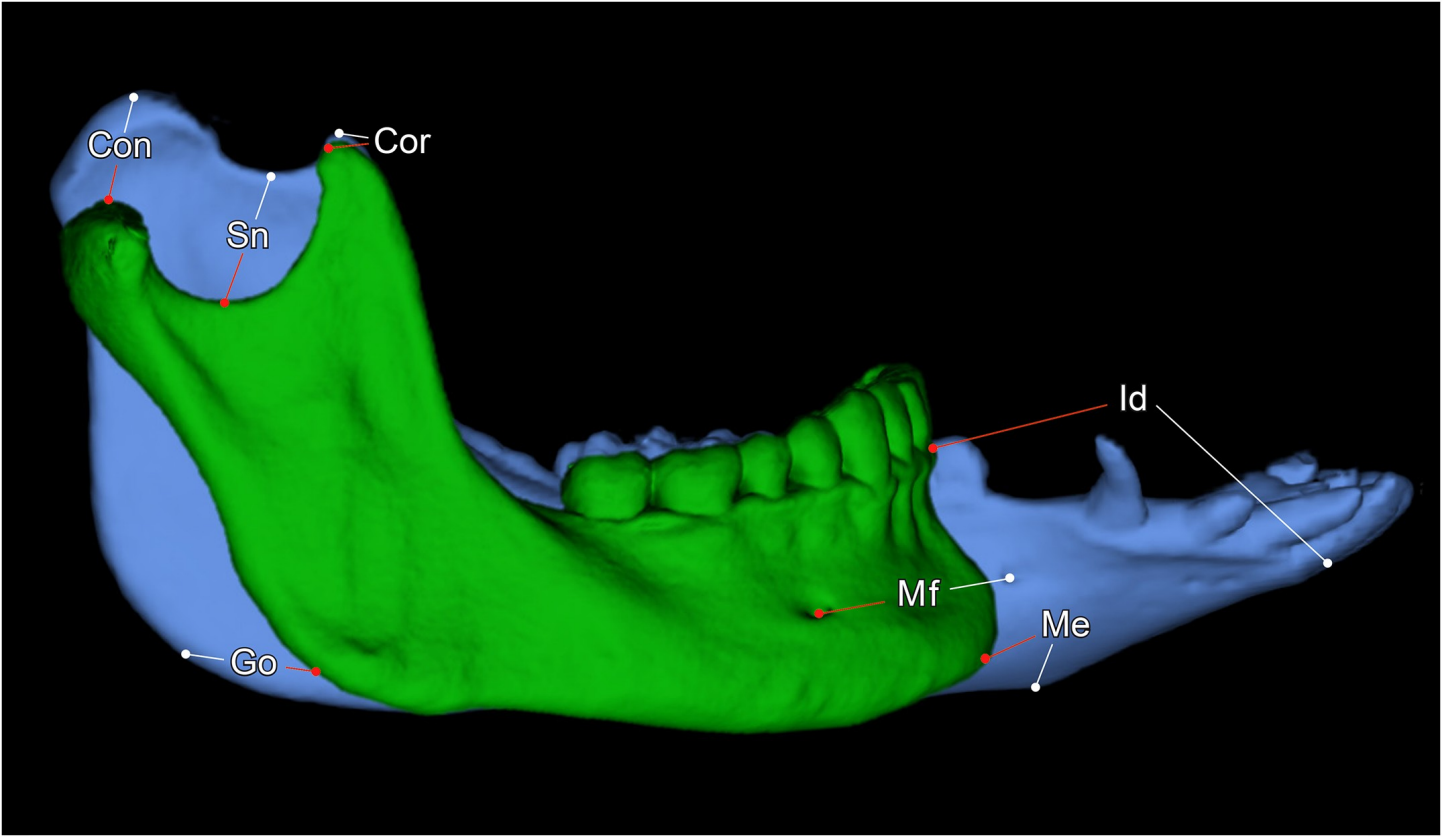
The different morphology of the minipig and human mandible. 3D renderings of an adult human mandible (green) and a mandible of a 21-months old Göttingen Minipig. Both segmentations are presented at the same scale. Where: Con = condylion, Cor = coronion, Sn = lowest point of the sigmoid notch, Go = gonion, Mf = posterior prominent mental foramen, Me = menton, Id = infradentale.

Fig 9 shows the elongate mandible of minipig and its anteriorly directed mentum. Humans have a much shorter mandible and a more vertical mentum, with an anteriorly located menton. Minipigs have a longer and steeper mandibular ramus with a longer and larger mandibular condyle. Their coronoid process and mandibular condyle are approximately located at the same height. Humans have a more elongate and deeper sigmoid notch as well as an inferiorly located mandibular condyle in relation to the coronoid process.

### The position and dimensions of the masticatory muscles

**Fig 10:**
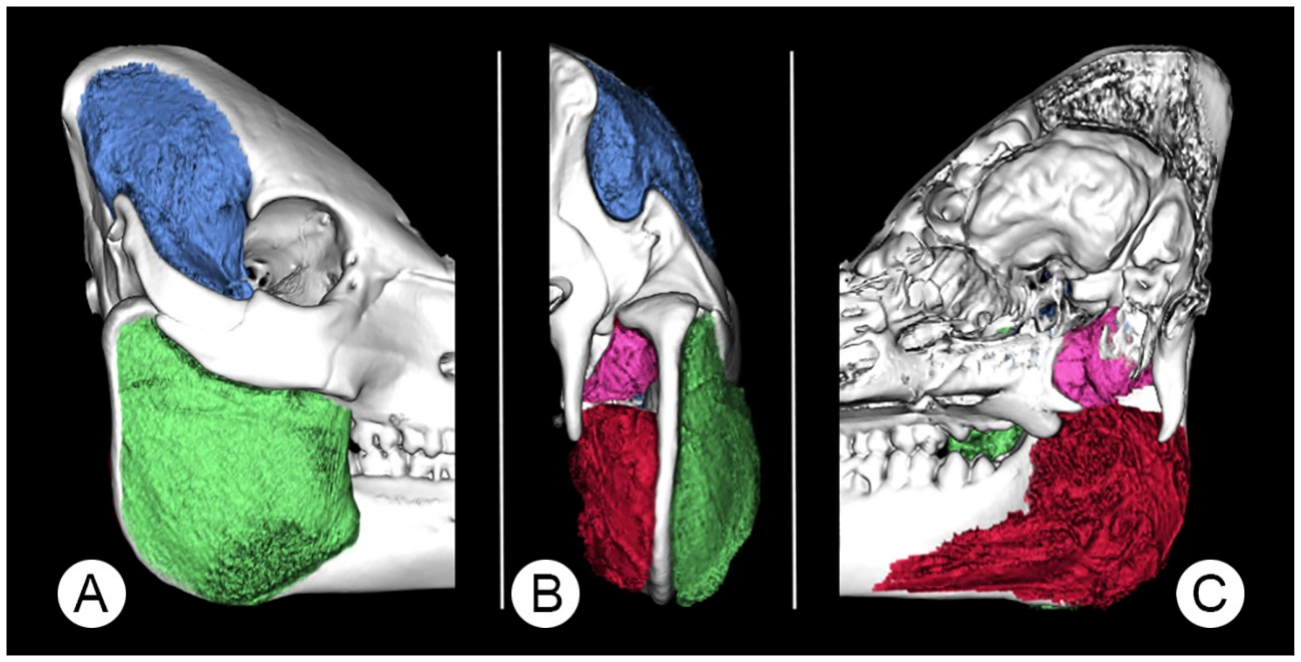
A 3D-rendered skull of a 12 months-old Göttingen Minipig showing the segmented masticatory muscles. Where (A) is a lateral, (B) a posterior and (C) a medial view. Pictured are the masseter (green), temporal (blue), medial pterygoid (red) and lateral pterygoid (pink) muscles.

In the minipigs, the masseter muscle (Fig 10A and 10B) is a nearly square shaped, thick muscle that originates from the inferior aspect of the facial crest, the complete inferior aspect of the zygomatic arch and the lateral aspect of the mandibular process, directly inferior to the mandibular condyle. Its insertion is the mandibular body extending from the vertical at the level of the distal aspect of the second molar tooth (M2) through to the posterior border of the mandibular ramus.

The temporal muscle (Fig 10A and 10B) is much thinner than the masseter muscle. It originates from the temporal fossa, terminating anteriorly at the level of the zygomatic process of the frontal bone and posteriorly adjacent to the nuchal line and supramastoid crest. The temporal muscle also originates from the superior aspect of the zygomatic process of the temporal bone. The temporal muscle inserts on the coronoid process and the anterior aspect of the mandibular ramus, in close proximity with both pterygoid muscles.

The pterygoid muscles consist of a large medial muscle block and a smaller lateral muscle block. The inferior alveolar nerve passes between these to traverse the mandibular foramen into the mandibular canal.

The medial pterygoid muscle (Fig 10B and 10C) originates from the inferolateral aspect of the pterygoid bone, the pterygoid hamulus and the sphenoidal process of the palatal bone. It travels in close proximity to the tympanic bulla to its insertion at the lateral and posterior borders of the mandibular ramus. An inferior portion extends across the medial aspect of the mandibular body as far anteriorly as the second premolar tooth.

The lateral pterygoid muscle (Fig 10B and 10C) originates from the dorsolateral aspect of the pterygoid bone and the dorsal aspect of the pterygoid hamulus. Its insertion is directly beneath the medial aspect of the mandibular condyle.

### Blood vessel architecture adjacent the mandibular ramus

**Fig 11:**
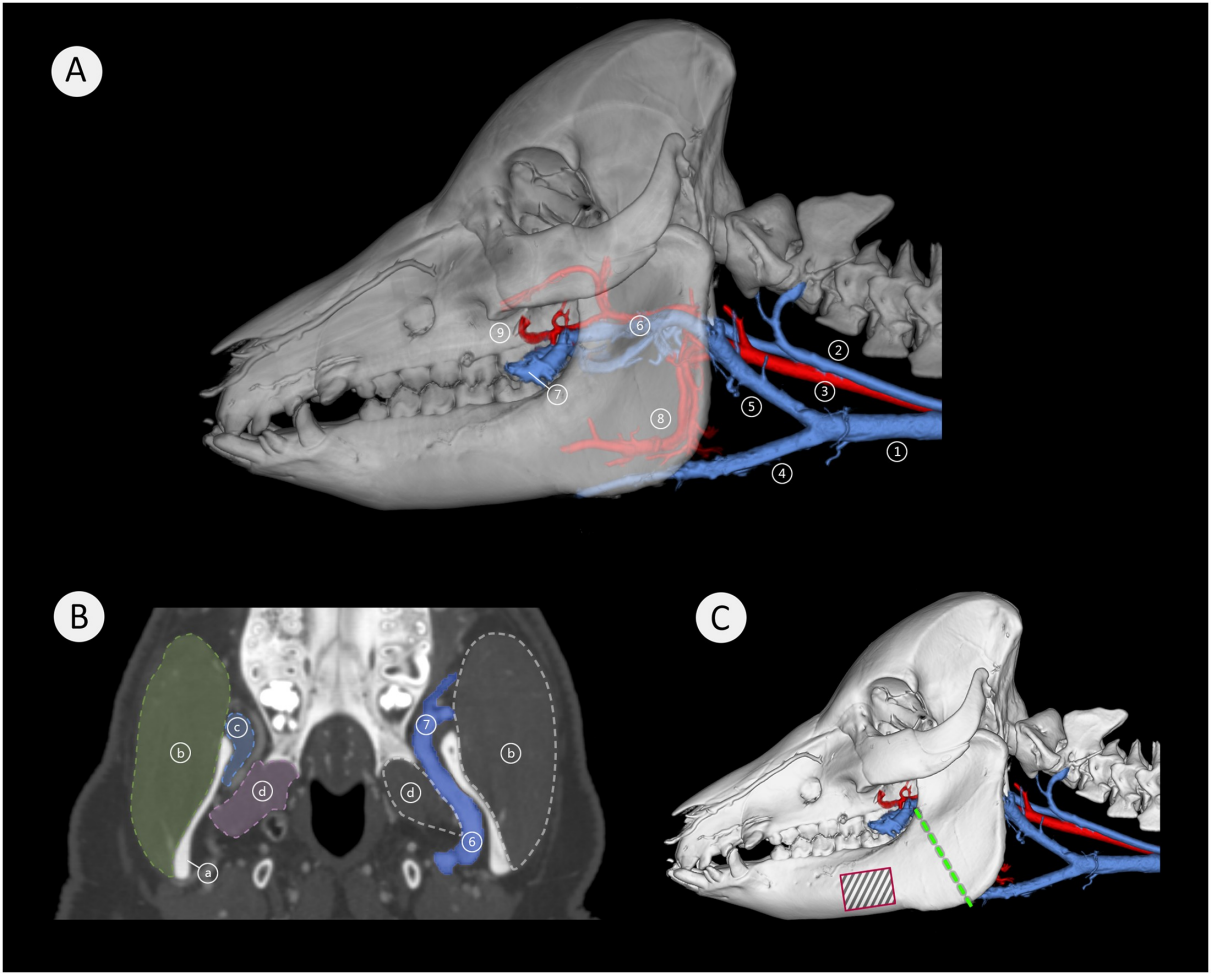
Vascular architecture medial to the mandibular ramus. Image (A) is a lateral view of a semitransparent segmentation of a 21 months-old minipig head with associated major blood vessels of the neck and the mandibular region. Arteries are pictured in red and veins in blue. Here: (1) external jugular vein, (2) internal jugular vein, (3) common carotid artery, (4) linguofacial vein, (5) maxillary vein, (6) deep facial vein with maxillary artery, (7) deep facial vein traversing from medial to lateral, (8) lingual artery, (9) buccal artery. Image (B) shows a coronal view with the prominent deep facial vein (6) (in blue), adjacent to the medial aspect of the mandibular ramus (a). The vein has a diameter of approximately 6 mm and traverses from medial to lateral across the anterior aspect of the mandible (7). Here; (a) mandibular ramus, (b) masseter muscle, (c) temporal muscle insertion, (d) lateral pterygoid muscle, (6) deep facial vein, (7) deep facial vein traversing from medial to lateral. Image (C) is a lateral view of a 21 months-old minipig skull with associated large blood vessels of the neck and the mandibular region. Arteries are pictured in red and veins in blue. The green dashed line indicates the most common sectional plane used in experimental mandibular distraction osteogenesis procedures, the black-striped red rectangle indicates a common site for fixation plate placement in some experimental surgery (Fig 12B and 12C).

The 3D rendering of the blood vessel architecture shows that both the maxillary artery and the deep facial vein (V. faciei profunda) lie in close proximity to the medial aspect of the mandibular ramus. The deep facial vein originates from numerous slender superficial facial veins immediately anterior to the mandibular ramus. From here it dives around the anterior edge of the mandibular ramus, to run posteriorly immediately adjacent the mandibular ramus. At this level, it has a diameter of approximately 6 mm. It then drains posteriorly into the maxillary vein. The deep facial vein is accompanied by the maxillary artery as it traverses medial to the mandibular ramus. Inferior to the maxillary artery and the deep facial vein runs the lingual artery along its arcuate course (Fig 11A).

The two-dimensional coronal plane image (Fig 11B) shows the horizontally running deep facial vein and its mediolateral course around the anterior aspect of the mandibular ramus. The portion of the vein medial to the ramus has a diameter of approximately 6 mm.

### Theoretical space available for mono- and bicortical screw insertion

**Fig 12:**
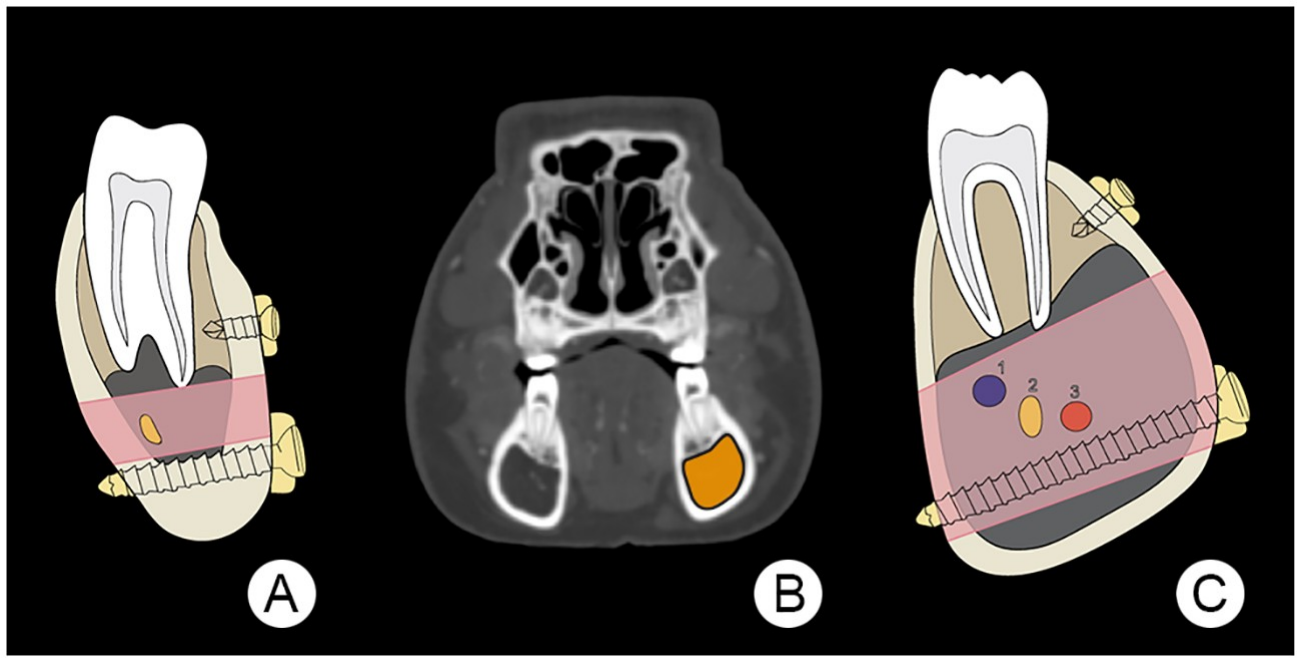
Illustration of theoretical space available for mono- and bicortical screw insertion. Here (A) is an illustration of the human mandibular body (after the AO Foundation, Switzerland), showing the potential space for positioning both mono- and bicortical screws. The pink area indicates a zone, which extends from the tooth roots to the inferior aspect of the mandibular canal that conveys the inferior alveolar nerve and its associated blood vessels. The yellow oval indicates the inferior alveolar nerve. Image (B) is a transverse plane CT image of a 21 months-old minipig head at the level of the first premolar tooth. The area coloured in orange indicates the dimensions of the mandibular canal. Illustration (C) depicts the right mandibular body of the minipig seen in (B), showing one extreme of the highly variable mandibular canal dimensions and the potential space for positioning both mono- and bicortical screws. The pink area indicates a zone where the inferior alveolar nerves and blood vessels are located. Portrayed is the inferior alveolar neurovascular bundle consisting of the inferior alveolar vein (1), the inferior alveolar nerve (2) and the inferior alveolar artery (3).

The illustration of the human mandibular body (Fig 12A) demonstrates the correct positioning of mono- and bicortical screws in order to avoid damage to the tooth roots and the inferior alveolar neurovascular bundle. The minipig shown in Fig 12B, has a large mandibular canal volume [17]. Compared to the human (Fig 12A), the inferior mandibular bone thickness of the minipig is notably thinner as are the buccal and lingual cortices of the mandibular body (Fig 12C). In addition, the shape of the mandibular body in both species clearly differs greatly with each other. Whilst the mandibular body cross section of humans is usually ovoid in shape (Fig 12A), that of minipigs is highly variable, ranging from ovoid to pear-shaped (Fig 12C). In some minipigs, the most inferior point of the mandibular body can be located at the lingual side of the body, whereas the most buccal point is more or less located on a horizontal midline through the center of the mandibular canal.

## Discussion

In presurgical planning of human mandibular surgery and reconstruction, numerous cephalometric parameters are measured routinely. Because experimental approaches for these procedures are often developed in Göttingen Minipigs, we selected 22 of these parameters and measured them using CT scans of subadult and adult Göttingen Minipigs. By doing so, we evaluated the dimensions and the overall anatomical growth changes and ultimately compared these with human data from the literature. Of the 22 parameters measured only four were found to be highly comparable, whilst the others were not.

These four parameters were the distance from the mental foramen vertically to the mandibular alveolar crest (MAC), the distance from mental foramen to the gonion (MGO), the mandibular condyle volume (MCV) and the intergonial breadth (IGB). They all had a MP:H between 0.9 and 1.1 in at least one age group (Table 5).

In the present study, the MAC in minipigs decreased from 13.4 mm at 12m to 10.6 mm at 17m but not thereafter. Comparably in humans, Ozturk et al. (2013) reported a MAC of 11.4 mm [60]. In another study on 307 human patients, a mean MAC of 11.84 ± 3.02 mm was reported [71]. Compared to humans, especially older minipigs of the 17m and 21m group, showed a high comparability, indicating that in these age groups, the position of the mental foramen in relation to the alveolar crest is very similar.

In the minipigs of the present study, the second highly comparable feature, i.e. the distance from the mental foramen to the gonion, increased significantly with age. At 12m it was 81.6 mm and by 21 m it was 90.9 mm. Tebo and Telford (1950) reported a MGO in humans of 74.6 mm, which is highly comparable to values found in 12m minipigs [70].

The third highly comparable parameter, the mandibular condyle volume, in the minipigs ranged from 1.9 ml at 12m to 2.7 ml at 17 m and 2.9 ml at 21m. However, there were large individual differences within each age group. As an example, the 21 months group showed values ranging from 1.4 ml to 3.8 ml. In a volumetric assessment of 700 human mandibular condyles, Safi et al. (2017) reported a mean MCV of 2.44 ml in the right and 2.27 ml in the left condyle [77]. Similarly Saccucci et al. (2012) reported a mean MCV of 2.7 ± 0.5 ml for the right and 2.7 ± 0.4 ml for the left condyle in 65 adolescent human patients [78]. This indicates that the MCV of 17m old minipigs is highly comparable to that of humans.

The fourth highly comparable parameter was the intergonial breadth that in minipigs ranged from 97.0 mm at 12m to 111.8 mm at 21 m. In the present study, only the 12m old animals’ parameters were highly comparable with humans. In humans, Weijs and Hillen (1984) reported an IGB of 107.0 ± 5.0mm [64]. Steyn and Iscan (1998) presented an IGB of 99.6 ± 5.5 mm in males and 91.5 ± 5.0 mm for females [63]. Similarly a Brazilian study from 2013 reported that the IGB ranged from 93.7 ± 6.8 mm to 94.5 ± 9.1 mm [79]. More recently Lopez et al. (2017) reported an IGB of 91.8 ± 5.9 mm for males and 84.5 ± 5.0 mm for females [57]. Ozturk et al. (2013) published an IGB of 85.86 mm [60]. Thus whilst the IGB of the 12m minipigs has a high comparability with humans that of older minipigs has not.

The lingual intercrestal breadth (LIB), the interforaminal breadth (IFB) and the mandibular ramus length (MRL) showed moderate comparability in at least one minipig age group with published data for humans (MP:H between 0.85 and 1.15) (Table 5).

The LIB, a rough indicator of the intercanine width, in minipigs ranged from 28.3 mm at 12m to 30.4 mm at 21m. Bishara et al. (1997) reported the intercanine width to be 25.4 ± 1.8 mm in 13 year old and 24.4 ± 1.4 mm in 26 year old human females [67] whilst Tamewar and Parakh (2018) reported it to be 26.4 ± 2.9 mm in adolescents [69] and Singh et al. (2017) found it to be 25.3 ± 0.9 mm in 209 females [68]. As seen in MGO and IGB, especially the younger groups of minipigs had comparable values and therefore similar dimensions in this region.

In minipigs, the IFB ranged between 51.6 mm at 12m to 57.9 mm at 21m. The closest comparability was between 12m old minipigs and humans. The older minipigs showed no comparability. In humans, Lopez et al. reported (2017) an IFB of 46.5 ± 3.7 mm for males and 44.6 ± 2.5 for females, Kumar and Lokanadham (2017) reported an IFB of 43.2 ± 2.8 mm [72] and Dong et al. (2015) an IFB of 49.93 ± 3.01 mm in males and 47.23 ± 2.80 mm in female individuals [73]. Hence, the IFB of 12m old minipigs is comparable to that of humans; the older age groups are not comparable.

The mandibular ramus length remained at 42.3 mm in 12 and 17 months old minipigs but then increased to 44.5 mm by 21m. In humans, Kim et al. (1997) reported a mean MRL of 32.7 ± 2.8 mm [62], whilst Giles (1964) reported an MRL of 39.84 ± 3.69 mm in males and 37.83 ± 2.93 mm in females [74]. When compared to 12m and 17m minipigs the MRL of humans had a moderate level of comparability.

The 11 remaining parameters showed no comparability between Göttingen Minipigs and humans, as they had a MP/H-ratio <0.85 and >1.15in in all three age groups.

1. MRH: Compared to the published values of humans, Göttingen Minipigs have a significantly higher mandibular ramus [57–60].
2. oMRH: The oblique mandibular ramus height is significantly higher in Göttingen Minipigs than in humans [61].
3. iMBL: In minipigs, the infradentale is the most anteriorly located mandibular point and contributes to the overall length of the mandible. Minipigs possess a significantly longer mandible than humans [58, 59, 63, 64].
4. DAL: The presence of a diastema in minipigs prevents a reasonable comparison with humans but served as an anteroposterior growth indicator. Our data show, that the premolar and molar dental arch length is longer in minipigs than the overall dental arch length in humans, measured from central incisors to the last molar tooth [65, 66].
5. MIB: In minipigs the MIB is nearly twice that of humans [60]. The mental foramen in humans is in the vertical center of the mandibular body, whilst in minipigs, the mental foramen has a more superior position, relative to the mandibular body height.
6. GA: Measurements of the gonial angle revealed that minipigs have a much more oblique mandibular angle compared to humans [57, 59, 64, 73]. In humans, the most posterior point of the mandible is the mandibular condyle whilst in minipigs it is the posterior ramus edge above the gonion (Fig 9).
7. SRL: Minipigs have a shorter superior ramus length than humans [57, 75].
8. CPV: The coronoid process volume of minipigs is not comparable to that of humans who have a significantly higher volume [76]. Noteworthy is the high individual variation in minipigs of the same age. For example in the 21m group, the lowest volume was 53.8 mm³ whilst the highest was 399.2 mm³.
9. AMH: Minipigs have a higher anterior mentum height compared to human values reported in the scientific literature [60, 72, 74]. This is because minipigs do not have a posteriorly located infradentale as found in humans.
10. ICB: Because of the smaller area between their superior mandibular ramus, minipigs have a lower intercondylar breadth (ICB) than humans [57, 63, 72].
11. ICOB: Of the three parameters (ICOB, ICB and SNB) which assess laterolateral growth between the superior ramus of both hemimandibles, only the ICOB had statistically significant ongoing changes with age. This indicates that the superior ramus width does change in the anterior region between the coronions and remains constant in the posterior region.

Four parameters, namely the mandibular body length (MBL), the diastemal length (DL), the intercrestal breadth (IB) and the breadth between sigmoid notches (SNB), could not be compared, because to the best of our knowledge, there is no data reported on humans (Table 5). Mandibular body length of minipigs increased steadily with age. Whilst the presence of a diastema in the minipigs prevents a direct comparison with humans, increases in minipig diastemal length indicate longitudinal growth. However, in our study there were no significant changes in DL over time. This suggests that the major part of anteroposterior mandibular growth occurs in the posterior ramus area. Studies conducted on mandibular growth in humans and pigs confirm this observation [80–82].

In this study, the 3D segmentations show that the growth changes of the whole mandible, the mandibular condyles and the superior mandibular ramus between minipigs of 17 and 21m, corresponded to the cephalometric measurements undertaken in this study.

The quadrupedal mode of life has a significant influence on the architecture and distribution of the vasculature of the head and neck when compared to that of bipedal humans. In a quadruped at the transition of the neck to the head the vasculature courses in a horizontal, posteroanterior manner, whilst that of bipeds is vertically directed [20, 21]. The 3D examinations of the minipig vasculature showed an extensive, large, tortuous network of veins and to a lesser extent arteries immediately medial to the mandibular ramus (Fig 11). The very prominent, deep facial vein and maxillary artery form a deep facial vascular complex that has not been reported previously and is potentially important to experimental MDO procedures in Göttingen Minipigs. Commonly the principal sectional plane for MDO procedures extends from the inferior border anterior to the mandibular angle to the retromolar region [83, 84]. In Göttingen Minipigs, the presence of the deep facial vascular complex adjacent to where the mandible is sectioned, constitutes a major risk factor. Any accidental transection of these blood vessels could result in uncontrollable inaccessible bleeding. Whilst the lingual artery and linguofacial vein could potentially interfere with the MDO sectional plane their more medial location makes them less vulnerable.

As illustrated in Fig 12 the morphology and dimensions of the mandibular body in humans and minipigs are very different. Whilst humans have a mandibular body with an ovoid cross-section (Fig 12A), that of minipigs can be pear-shaped (Fig 12C). In a previous study we showed large individual differences in the dimension of the mandibular canal of Göttingen Minipigs of the same age [17]. Minipigs also have a significantly thinner inferior mandibular body bone thickness (4.7 mm at 12m and 4.0 mm at 21m) than humans (9.4 mm to 12.6 mm) [17, 85, 86]. Consequently, bicortical screws that are positioned in the inferior part of the mandibular body routinely in humans could, when placed in a similar way in a Göttingen Minipig, cause trauma to the inferior alveolar nerves and vessels. This could be compounded by the erratic highly variable position of the inferior alveolar nerves and vessels with their possible undulating course, often resembling a corkscrew [17]. Bicortical screws implanted in the inferior cortex would probably, due to the thin inferior bone thickness, have impaired stability.

The segmentation of the masticatory muscles of the minipigs revealed similar findings to that reported in the literature on larger domestic pig breeds. However, we found that the masseter muscle of Göttingen Minipigs extended more anteriorly than previously described [21]. When compared with humans, minipigs have a larger masseter but smaller temporal muscle. Whilst the lateral pterygoid muscle of the minipig has a comparable anatomical position and dimension to that of humans, the medial pterygoid muscle is larger and has a similar origin, but its insertion is located far more anteriorly. It extends to the height of the first molar tooth [84, 87–89]. Herring et al. observed that the dynamics of mastication in pigs and in humans differ greatly. Under natural conditions, pigs have a rapid rate of mastication and each side of the dental arcade is used independently. Contrary to this, humans have a slower and unilateral mastication. In addition, pigs have a higher crushing force and closing velocity than humans, that could potentially impair wound healing and implant stability [20]. An additional negative influence potentially promoting these post-operative complications often observed by surgeons undertaking mandibular surgical procedures, is that post-operatively pigs grind their teeth extensively as well as bite hard objects such as their cages [20, 37, 90, 91].

In 2002, Swennen et al. stated, “that appropriate animal models would be those that exhibit similar regional growth vectors and patterns to humans. Because it is obvious that a single animal model cannot be appropriate for all craniofacial regions, fitting appropriate animal models should be based on comparative data of anatomical characteristics and growth patterns of the craniofacial region of interest and the expected level of extrapolation to the human clinical condition, rather than on the phylogenetic affinity” [92]. Our study corroborates Swennen’s observations. We found significant differences in the mandibular anatomy of minipigs compared to data of humans. This raises concerns, that extrapolating acquired scientific results of Göttingen Minipigs to humans could be misleading or incorrect. This in turn suggests that Göttingen Minipigs are not ideal for experimental mandibular surgery research. Due to the lack of alternative large animal models, the authors recommend to precisely plan mandibular surgical experiments based on radiographic techniques, such as Computed Tomography, and to choose suitable age groups and use customized implants based on the mandibular dimensions as reported in this study.

## Conclusions

Based on the results of this study, the authors consider the Göttingen Minipig not to be an anatomically ideal animal model for experimental mandibular surgery research. The minipig mandible not only differs greatly from that of humans but also is highly variable in its morphology within animals of the same age group. This in fact requires carefully conducted presurgical planning using radiographic techniques, such as Computed Tomography. The minipig mandibular anatomy of younger animals (12m) is aligned more closely to that of humans. However, because of ongoing growth changes until the age of 21 months, only older Göttingen Minipigs should be used. The anatomical properties of mandible of the minipigs, i.e. the blood vessels medial to the ramus interfering with the sectional plane for MDO, can result in complications that are relevant to animal welfare and may additionally contribute negatively to their suitability.

## Supporting information

S1 Dataset Table with results of all measured parameters of the different age groups of Göttingen Minipigs. (XLSX)
